# Prolonged Boarding and Racial Discrimination and Dissatisfaction Among Emergency Department Patients

**DOI:** 10.1001/jamanetworkopen.2024.33429

**Published:** 2024-09-16

**Authors:** Rose McKeon Olson, Andrea Fleurant, Sophie Grace Beauparlant, DaMarcus Eugene Baymon, Regan Marsh, Jeffrey Schnipper, Marie Plaisime, Bram Wispelwey

**Affiliations:** 1 Division of Global Health Equity, Department of Medicine, Brigham and Women’s Hospital, Boston, Massachusetts; 2 Harvard Medical School, Boston, Massachusetts; 3 Department of Emergency Medicine, Brigham and Women’s Hospital, Boston, Massachusetts; 4 Hospital Medicine Unit, Division of General Internal Medicine and Primary Care, Brigham and Women’s Hospital, Boston, Massachusetts; 5 FXB Center for Health and Human Rights, Harvard T.H. Chan School of Public Health, Boston, Massachusetts

## Abstract

**Importance:**

Emergency department (ED) boarding times have increased rapidly, but their health equity outcomes are unknown.

**Objective:**

To investigate whether prolonged ED boarding is associated with increased perceived racial discrimination and dissatisfaction and whether associations vary between patients from marginalized racial and ethnic groups vs non-Hispanic White patients.

**Design, Setting, and Participants:**

This is a cross-sectional study of hospitalized adults who boarded in the ED during internal medicine admissions at a large, urban hospital in Boston, Massachusetts, from June 2023 to January 2024. Equal proportions of non-Hispanic White patients and patients from marginalized racial and ethnic groups (American Indian or Alaska Native, Hispanic, non-Hispanic Black and/or African American, and multiracial) were selected randomly.

**Exposure:**

The duration of ED boarding was categorized as less than 4 hours (reference), 4 to less than 24 hours, and 24 or more hours.

**Main Outcomes and Measures:**

Primary outcomes were odds of reporting (1) discrimination via the Discrimination in Medical Settings scale, and (2) dissatisfaction via the adapted Picker Patient Experience–15 questionnaire. Marginalized race and ethnicity was tested as an effect modifier. Multivariable logistic regression models adjusted for patient age, sex, language, and insurance payer.

**Results:**

Of 598 patients approached, 527 were enrolled, and 525 completed the surveys (response rate, 87.8%). The mean age (SD) was 60.6 (18.7) years, 300 patients (57.1%) were female, 246 patients (47.3%) identified as non-Hispanic White, and 274 (52.7%) were from a marginalized racial or ethnic group. In total, 135 (25.7%) boarded less than 4 hours (reference), 202 (38.5%) boarded 4 to less than 24 hours, and 188 (35.8%) boarded 24 hours or longer. Compared with less than 4 hours, boarding 24 hours or longer was associated with increased perceived discrimination (odds ratio [OR], 1.84; 95% CI, 1.14-2.99; *P* = .01). An increased association was observed in the subgroup of patients from racial and ethnic marginalized groups (OR, 2.36; 95% CI, 1.20-4.65; *P* = .01); effect modification was not significant (*P* for interaction, .10). For all patients, boarding 24 hours or longer was associated with increased dissatisfaction with care (OR, 1.77; 95% CI, 1.03-3.06; *P* = .04); effect modification was not significant (*P* for interaction, .80).

**Conclusions and Relevance:**

In this cross-sectional study, hospitalized patients who boarded in the ED 24 hours or longer reported more discrimination and dissatisfaction with care, which may disproportionately affect patients from marginalized racial and ethnic groups. As ED boarding times increase nationally, it is critical to recognize their potential to exacerbate health inequities and to respond with equity-focused solutions.

## Key Points

**Question:**

Is prolonged emergency department (ED) boarding associated with racial discrimination and dissatisfaction?

**Findings:**

In a cross-sectional study of 525 adults admitted to a large, urban medical center in Boston, Massachusetts, patients who boarded 24 hours or longer were 1.84 times more likely to report discrimination and 1.77 times more likely to report dissatisfaction with care, compared with those who boarded less than 4 hours.

**Meaning:**

These findings suggest that patients who board in the ED 24 hours or longer may experience more racial discrimination and dissatisfaction with care, which may exacerbate preexisting health inequities.

## Teaser

This cross-sectional study examines whether prolonged emergency department (ED) boarding is associated with increased perceived racial discrimination and patient dissatisfaction.

## Introduction

Emergency department (ED) boarding time has been rapidly increasing across the US and has been declared a national public health crisis by the American College of Emergency Physicians.^1,2,3^ Exacerbated by the COVID-19 pandemic, increased ED boarding has led to capacity challenges that place patients at higher risk for harm, including increased mortality, intensive care unit admissions, length of stay, and medical errors.^4,5,6,7^ ED boarding refers to patients who have been admitted to the hospital but physically remain in the ED owing to a lack of available inpatient beds. The Joint Commission identified boarding as a patient safety risk that should not exceed 4 hours, yet the proportion of patients boarding 24 hours has more than doubled from 2018 to 2020.^2,8,9^ Despite these known adverse outcomes, the impact of increasing ED boarding times on patients from marginalized racial and ethnic groups is unknown. Although prior studies have linked lower patient satisfaction to longer ED boarding,^10^ there is a gap in data since the onset of the COVID-19 pandemic and the parallel surge in boarding.

This study was designed acknowledging race as a ubiquitous and potent force in society and, therefore, in health care.^11,12^ We understand race as a social construct that creates and maintains human hierarchy; relevant differential outcomes, therefore, reflect the impacts of racism.^11,12^ As structural and institutional racism are often exacerbated by environmental stressors and limited resources, we hypothesized that the overcrowding and capacity constraints associated with prolonged boarding may have a disproportionate effect on patients from marginalized racial and ethnic groups. The aim of our study was to determine whether ED boarding time is associated with (1) increased perceived racial and ethnic discrimination, and (2) increased patient dissatisfaction among patients admitted to internal medicine services.

## Methods

### Sample

This cross-sectional study was reviewed and approved by the Mass General Brigham institutional review board and follows the Strengthening the Reporting of Observational Studies in Epidemiology (STROBE) reporting guideline for cross-sectional studies.^13^ We conducted random-sample, in-person surveys of adult patients who presented to the ED and were admitted to internal medicine services at a large urban academic hospital in Boston, Massachusetts, from June 2023 to January 2024. The race and ethnicity listed in the electronic medical record (EMR) was used to facilitate random sampling to obtain 1:1 samples of (1) non-Hispanic White patients and (2) American Indian or Alaska Native, Hispanic, non-Hispanic Black and/or African American, and multiracial patients, hereafter referred to as patients from marginalized racial and ethnic groups. These groups were selected because local health inequities are known to primarily impact these groups.^14,15^ Verbal informed consent was obtained according to local institutional review board requirements. During enrollment, patients provided their self-reported race and ethnicity, which was subsequently used in all aspects of study reporting and analysis. We excluded direct admissions, interhospital and interservice transfers, intensive care unit admissions, and patients whom the primary medical team thought were inappropriate for enrollment. All surveys were conducted in person during the index hospitalization, after transfer from the ED to the inpatient medicine floors, and thus after the ED boarding had ended.

### Exposures of Interest

Boarding time was calculated according to Centers for Medicare & Medicaid Services guidelines as the median difference in hours and minutes between the decision for hospital admission to physical departure from the ED.^16^ Boarding time was first examined as a continuous variable, and a categorical variable was created: less than 4 hours (reference), 4 to less than 24 hours, and 24 hours or longer.^8^ The reference was selected according to the Joint Commission’s safety cutoff, and boarding for 24 hours or longer has been associated with adverse outcomes.^2,8,9,17,18^

### Outcomes

To assess experiences of discrimination, we used the 7-item Discrimination in Medical Settings (DMS) Scale, which is adapted from the Everyday Discrimination Scale for medical settings (eAppendix 1 in Supplement 1).^19,20^ We asked patients to reflect specifically on their experience while boarding in the ED. Participants who responded affirmatively to rarely, sometimes, most of the time, or always to any discrimination experience were classified as yes for the binary outcome. This classification was chosen to improve interpretability and because of rightward skew of responses, as done previously.^21,22,23^ For exploratory purposes, participants were asked whether, besides race and ethnicity, there were other aspects of their identity they felt were discriminated against during boarding. Participants could provide multiple responses.

The Picker Patient Experience Questionnaire (PPE-15) was adapted to measure patient experience and satisfaction while boarding in the ED (eAppendix 2 in Supplement 1).^24^ Questions 11, 12, and 15 were omitted from the adapted questionnaire because they were either discharge-related or time-dependent and likely to be associated with the primary exposure, length of boarding time. Similar to the DMS scale, responses from the PPE-15 had strong rightward skew, and thus were dichotomized according to the original PPE-15 problem score, where a problem is defined as, “an aspect of healthcare that could, in the eyes of the patient, be improved upon,” as done previously.^24,25^

### Potential Confounders and Effect Modifiers

A priori potential confounders are reported in Table 1 and included age, sex, primary language, and health insurance payer (additional details are shown in eTable 1 in Supplement 1). We assessed collinearity among all covariates using variance inflation factors and examined correlation matrices. The results indicated no evidence of substantial collinearity issues among the included variables. A priori, self-identified marginalized race and ethnicity was considered a potential effect modifier for both primary outcomes, with planned subgroup analyses.

**Table 1.** Respondent Characteristics

Characteristics	Participants, No. (%)						*P* value^c^
	All	Non-Hispanic White	Marginalized race and ethnicity^a^	Non-Hispanic Black	Hispanic	Other^b^	
Race and ethnicity	525 (100.0)	246 (47.3)	274 (52.7)	149 (28.7)	88 (16.9)	37 (7.1)	.22
Sex							
Female	300 (57.1)	139 (56.5)	159 (58.0)	87 (58.4)	51 (58.0)	21 (56.8)	.001
Male	223 (42.5)	107 (43.5)	113 (41.2)	62 (41.6)	36 (40.9)	15 (40.5)	
Age, mean (SD), y	60.6 (18.7)	63.0 (18.7)	58.6 (18.5)	59.2 (18.5)	55.7 (18.7)	62.9 (16.9)	.01
Language							
English	472 (90.1)	245 (100.0)	222 (81.0)	133 (89.3)	56 (63.6)	33 (89.2)	<.001
Spanish	33 (6.3)	0	33 (12.0)	1 (0.7)	32 (36.4)	0	
Haitian Creole	11 (2.1)	0	11 (4.0)	11 (7.4)	0	0	
Other	8 (1.5)	0	8 (2.9)	4 (2.7)	0	4 (10.8)	
Insurance							
Medicare	221 (42.1)	123 (50.0)	97 (35.4)	49 (32.9)	34 (38.6)	14 (37.8)	.001
Medicaid	44 (8.4)	12 (4.9)	32 (11.7)	12 (8.1)	14 (15.9)	6 (16.2)	
Private	248 (47.2)	108 (43.9)	136 (49.6)	85 (57.1)	35 (39.8)	16 (43.2)	
Other	12 (2.3)	3 (1.2)	9 (3.3)	3 (2.0)	5 (5.7)	1 (2.7)	
Means of arrival							
Ambulance	231 (44.0)	142 (57.7)	86 (31.4)	41 (27.5)	32 (36.4)	13 (35.1)	<.001
Car	223 (42.5)	76 (30.9)	146 (53.3)	83 (55.7)	42 (47.7)	21 (56.8)	
Other	71 (13.5)	28 (11.4)	41 (16.0)	25 (16.8)	14 (15.9)	3 (8.1)	
Boarding time, median (IQR), h	15.4 (3.8-32.7)	16.4 (3.6-33.9)	14.9 (3.9-31.1)	14.5 (4.1-28.7)	14.8 (3.7-34.9)	18.0 (6.1-31.1)	.74
Boarding time group							
<4 h (Reference)	135 (25.7)	64 (26.0)	69 (25.2)	37 (24.8)	23 (26.1)	9 (24.3)	.93
4 to <24 h	202 (38.5)	93 (37.8)	108 (39.4)	63 (42.3)	33 (37.5)	12 (32.4)	
≥24 h	188 (35.8)	89 (36.2)	97 (35.4)	49 (32.9)	32 (36.4)	16 (43.2)	

^a^ Includes self-reported marginalized race and ethnicity exclusive of non-Hispanic White.

^b^ Self-identified marginalized race and ethnicity includes Asian, Hispanic, Native American or Alaska Native, non-Hispanic Black and/or African American, and multiracial. See eTable 1 in Supplement 1 for additional details.

^c^ For non-Hispanic White vs marginalized race and ethnicity.

### Statistical Analysis

Respondent characteristics are reported overall, by major race and ethnicity group (ie, non-Hispanic White vs marginalized race and ethnicity), and by individual racial and ethnic subgroups. We compared sociodemographic characteristics, including sex, age, primary language, and health insurance payer. We calculated frequencies and proportions for categorical variables and means and SDs for continuous variables. We assessed associations with categorical variables using χ^2^ tests, associations with continuous normal variables with 2-sided *t* tests, and continuous nonnormal variables with Wilcoxon rank sum tests. Rates of missing data were low (<5% of observations). Two surveys were prematurely terminated and considered missing data. Missing data patterns were reviewed analytically and visually and were found to be missing at random. Given this, we performed a complete case analysis, wherein observations with missing data were excluded from analyses.

Using bivariable and multivariable logistic regressions, we estimated unadjusted and adjusted odds ratios (ORs) and 95% CIs to examine associations between length of boarding time by categorical level and reported experiences of discrimination and patient dissatisfaction, respectively. In the adjusted analyses, we controlled for age as a continuous variable, and sex, primary language, and insurance type as categorical variables. To investigate whether marginalized race and ethnicity was an effect modifier for both outcomes, we created an interaction term between the marginalized race and ethnicity and boarding time in our regression models. For interpretability, we also analyzed both outcomes by racial and ethnic subgroups. Next, we compared reported reasons for experiences of discrimination and dissatisfaction with care by racial and ethnic group and calculated frequencies and proportions (eTables 2 and 3 in Supplement 1). Because data were nonnormally distributed by both histogram and Shapiro-Wilk test, we used the Wilcoxon rank sum score to assess associations.

We conducted sensitivity analyses for race and ethnicity as categorical and binary variables (eTables 4 and 5 in Supplement 1). Analyses were limited to non-Hispanic-White, non-Hispanic-Black, and Hispanic groups because of low sample sizes in other categories. We defined statistical significance as *P* < .05 in 2-tailed tests. In subgroup analyses, we applied the Benjamini-Hochberg correction with a threshold of .20 for multiple comparisons. All analyses were conducted with Stata statistical software version 17.0 (StataCorp).

## Results

Of 598 patients approached, 527 were enrolled, and 525 completed the surveys, yielding a response rate of 87.8%. Among the 525 participants, the mean age (SD) was 60.6 (18.7) years, and 300 (57.1%) were female. Overall, 246 patients (47.3%) identified as non-Hispanic White, and 274 (52.7%) identified as being from a marginalized racial or ethnic group (Table 1). The median (IQR) boarding time was 15.4 (3.8-32.7) hours. There were 135 participants (25.7%) who boarded less than 4 hours (reference group), 202 (38.5%) who boarded 4 to less than 24 hours, and 188 (35.8%) who boarded 24 hours or longer. Respondents who identified as being from a marginalized racial and ethnic group were younger compared with non-Hispanic White participants, and fewer spoke English as a primary language. Fewer respondents from racial and ethnic marginalized groups had Medicare coverage and more had Medicaid coverage. More non-Hispanic White respondents presented by ambulance compared with respondents of marginalized racial and ethnic groups. There was no significant difference in boarding time by race and ethnicity.

Approximately one-third of respondents (170 of 525 respondents [32.4%]) reported they had experienced discrimination while boarding in the ED. Similar proportions of male (69 of 223 respondents [30.9%]) and female (100 of 300 respondents [33.3%]) patients reported discrimination. Younger patients reported higher proportions of discrimination, with a significant χ^2^ test for trend (*P* for trend, .001; aged 18 to <40 years, 38 of 98 patients [38.8%]; aged 40 to <65 years, 66 of 183 patients [36.1%]; aged 65 to <80 years, 46 of 164 patients [28.1%]; and aged ≥80 years, 19 of 78 patients [24.4%]). Compared with the non-Hispanic White group (71 of 246 patients [28.9%]), more patients from marginalized racial and ethnic groups (96 of 274 patients [35.0%]) reported experiencing discrimination while boarding. Higher rates of perceived discrimination were seen among non-Hispanic Black patients (56 of 149 patients [37.6%]) and other racial and ethnic identities combined (15 of 37 patients [40.5%]) compared with non-Hispanic White patients (25 of 88 patients [28.4%]).

More than three-fourths of patients (396 of 519 patients [76.3%]) reported dissatisfaction with at least 1 element of their care while boarding in the ED, with similar proportions among female (223 of 299 patients [74.6%]) and male (171 of 218 patients [78.4%]) patients. Younger patients reported higher proportions of dissatisfaction, with a significant χ^2^ test for trend (*P* for trend, .002; 82 of 98 patients [83.7%] aged 18 to <40 years, vs 146 of 181 patients [80.7%] aged 40 to <65 years, 114 of 161 patients [70.8%] aged 65 to <80 years, and 52 of 77 patients [67.5%] aged ≥80 years). More patients from marginalized racial and ethnic groups (215 of 273 patients [78.8%]) reported dissatisfaction compared with non-Hispanic White patients (176 of 241 patients [73.0%]). By subgroup, 115 of 149 (77.2%) non-Hispanic Black patients, 68 of 87 (78.2%) Hispanic patients, and 32 of 37 (86.5%) patients of other racial and ethnic identities reported dissatisfaction with care while ED boarding.

We observed statistically significant associations between length of boarding time with both perceived discrimination (Table 2) and dissatisfaction with care (Table 3). In the adjusted analyses, patients who boarded 24 hours or more were significantly more likely to report discrimination compared with patients who boarded less than 4 hours (OR, 1.84; 95% CI, 1.14-2.99; *P* = .01) (Table 2). In the subgroup of patients from racial and ethnic marginalized groups, boarding 24 or more hours was associated with increased discrimination compared with boarding less than 4 hours (OR, 2.36; 95% CI, 1.20-4.65; *P* = .01). The interaction between race and ethnicity and boarding time did not reach statistical significance (*P* for interaction, .10). Boarding times 4 to 24 hours compared with less than 4 hours were not significantly associated with discrimination for either respondents from marginalized racial and ethnic groups or non-Hispanic White respondents.

**Table 2.** Associations of Emergency Department Boarding Time With Experienced Discrimination, by Race and Ethnicity

Characteristic	Unadjusted OR (95% CI)	*P* value	Global *P* value	Adjusted OR (95% CI)^a^	*P* value	Global *P* value
Boarding time, h						
<4	1 [Reference]	NA	.01	1 [Reference]	NA	.01
4 to <24	0.98 (0.60-1.59)	.93		1.03 (0.63-1.69)	.90	
≥24	1.73 (1.08-2.79)	.02		1.84 (1.14-2.99)	.01	
Marginalized race and ethnicity^b^^,^^c^						
Boarding time, h						
<4	1 [Reference]	NA	.03	1 [Reference]	NA	.03
4 to <24	1.16 (0.59-2.26)	.67		1.33 (0.67-2.65)	.41	
≥24	2.18 (1.13-4.24)	.02		2.36 (1.20-4.65)	.01	
Non-Hispanic White^c^						
Boarding time, h						
<4	1 [Reference]	NA	.29	1 [Reference]	NA	.20
4 to <24	0.91 (0.44-1.89)	.80		0.95 (0.45-2.00)	.89	
≥24	1.48 (0.73-2.99)	.28		1.65 (0.79-3.29	.18	

Abbreviations: NA, not applicable; OR, odds ratio.

^a^ Adjusted for age, sex, language, and insurance type.

^b^ Includes Asian, Hispanic, Native American or Alaska Native, non-Hispanic Black and/or African American, and multiracial. See eTable 1 in Supplement 1 for additional details.

^c^ Adjusted *P* values reported according to the Benjamini-Hochberg correction for multiple comparisons.

**Table 3.** Associations of Emergency Department Boarding Time With Perceived Dissatisfaction, by Race and Ethnicity

Characteristic	Unadjusted OR (95% CI)	*P* value	Global *P* value	Adjusted OR (95% CI)^a^	*P* value	Global *P* value
Boarding time, h						
<4	1 [Reference]	NA	.06	1 [Reference]	NA	.02
4 to <24	0.92 (0.56-1.51)	.74		0.93 (0.56-1.54)	.77	
≥24	1.60 (0.93-2.73)	.09		1.77 (1.03-3.06)	.04	
Marginalized race and ethnicity^b^^,^^c^						
Boarding time, h						
<4	1 [Reference]	NA	.08	1 [Reference]	NA	.05
4 to <24	0.81 (0.40-1.64)	.48		0.80 (0.38-1.67)	.56	
≥24	1.79 (0.81-3.97)	.15		1.94 (0.86-4.40)	.11	
Non-Hispanic White^c^						
Boarding time, h						
<4	1 [Reference]	NA	.50	1 [Reference]	NA	.34
4 to <24	1.06 (0.53-2.15)	.86		1.13 (0.55-2.32)	.75	
≥24	1.48 (0.71-3.09)	.30		1.68 (0.79-3.60)	.18	

Abbreviations: NA, not applicable; OR, odds ratio.

^a^ Adjusted for age, sex, language, and insurance type.

^b^ Includes self-identified Asian, Hispanic, Native American or Alaska Native, non-Hispanic Black and/or African American, and multiracial. See eTable 1 in Supplement 1 for additional details.

^c^ Adjusted *P* values reported according to the Benjamini-Hochberg correction for multiple comparisons.

After adjusting for covariates, boarding 24 hours or longer significantly increased patient dissatisfaction compared with boarding less than 4 hours (OR, 1.77; 95% CI, 1.03-3.06; *P* = .04); effect modification was not significant (*P* for interaction, .80). Results were not significant for boarding 4 to 24 hours compared with less than 4 hours. In both prespecified subgroup analyses, the odds of patient dissatisfaction were not significantly different between patients from marginalized racial and ethnic groups and non-Hispanic White patients (Table 3). The interaction term between marginalized race and ethnicity and boarding time was not significant (*P *for interaction, .43).

**Figure 1.**
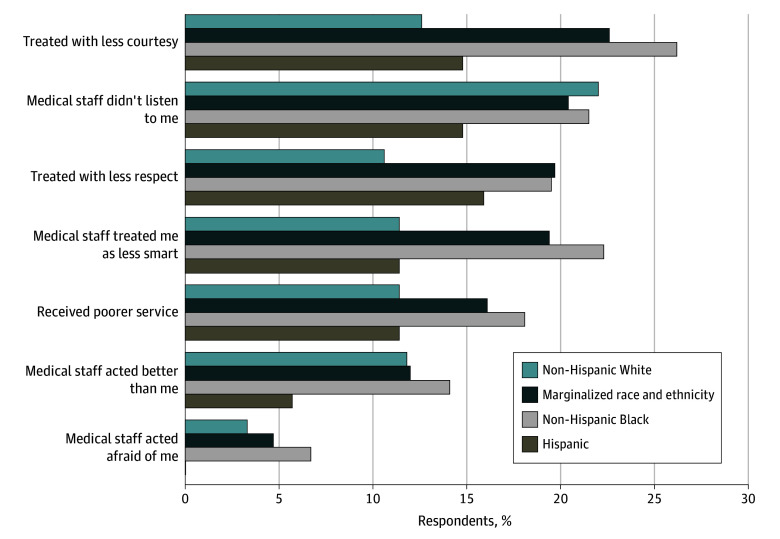
Reasons for Experiences of Discrimination During Emergency Department Boarding by Racial and Ethnic Group

The frequencies and proportions of various reasons for perceived discrimination, per the DMS scale, are illustrated in Figure 1 by racial and ethnic group. Patients from marginalized racial and ethnic groups were more likely to report being treated with less courtesy compared with non-Hispanic White patients (62 of 274 patients [22.6%] vs 31 of 246 patients [12.6%]), treated with less respect (54 of 274 patients [19.7%]) vs 26 of 245 patients [10.6%]), and treated as if they were less intelligent (53 of 273 patients [19.4%] vs 28 of 246 patients [11.4%]); these differences were attributed to the responses of non-Hispanic Black patients. See eTables 2 and 3 in Supplement 1 for additional details.

**Figure 2.**
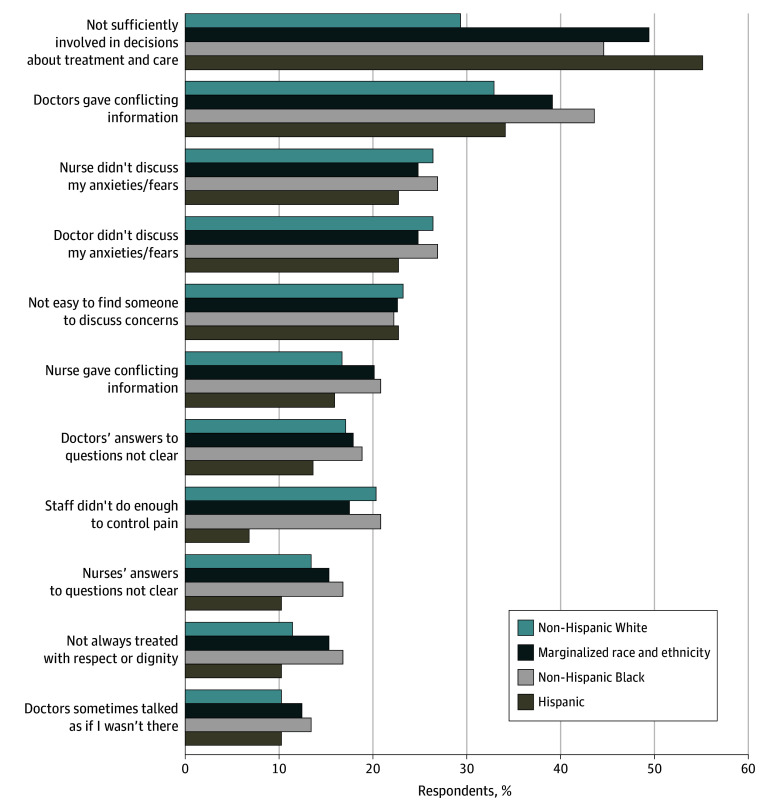
Reasons for Patient Dissatisfaction by Racial and Ethnic Group

The proportions and frequencies for dissatisfaction with care, via the adapted PPE-15, are presented in Figure 2 by racial and ethnic groups. Patients from marginalized groups were more likely to report not being sufficiently involved with decisions about their treatment or care compared with non-Hispanic White patients (128 of 274 patients [46.7%] vs 72 of 246 patients [29.3%]). Proportions and frequencies for other elements of dissatisfaction with care were similar between patients from marginalized groups and non-Hispanic White patients (Figure 2).

In exploratory analyses, patients were asked whether, besides their race and ethnicity, there were other aspects of their identity they felt were discriminated against during ED boarding. Of 82 responses, additional axes of discrimination included 9 for income or education level, 9 for shade of skin color, 8 for age, 7 for medical history, 7 for other aspect of physical appearance, 5 for gender, 5 for religion, 4 for physical disability, 4 for cultural or ancestral heritage, 3 for history of drug use, 2 for history of mental illness, 2 for sexual orientation, 1 for insurance type, 1 for homelessness, and 15 other (not otherwise specified).

## Discussion

This cross-sectional study of 525 hospitalized individuals admitted to internal medicine services evaluated whether longer ED boarding times were associated with perceived discrimination and dissatisfaction. Our results highlight 3 salient conclusions. First, prolonged ED boarding was common and associated with a threshold effect at 24 hours, at which point there was a significant increase in both perceived discrimination, particularly among patients from marginalized racial and ethnic groups, and dissatisfaction with care. Second, interactions with clinical staff may be an important factor underlying discrimination during ED boarding. Third, despite these findings, there remains a paucity of interventions aimed at reducing racial and ethnic inequities in prolonged ED boarding.

ED boarding 4 hours or longer is associated with increased patient harm.^8^ Notably, almost three-fourths of our sample boarded longer than 4 hours,^8^ and more than one-third boarded more than 24 hours, an amount of time that has been considered “an unconscionable occurrence.”^2^ As ED boarding increases and resources decrease, health care workers will face greater challenges in delivery of high-quality and equitable care.^7^ These alarming figures underscore the urgent need for systemic reforms and targeted interventions to address boarding challenges and safeguard patient well-being.

This study provides novel insight into racial health inequities linked to ED boarding. Our findings align with racial inequities seen in other consequences of ED overcrowding, including ED wait times, hallway bed use, triage, and discharges against medical advice.^26,27,28,29^ As boarding time is likely to increase alongside these factors, interventions to decrease boarding could have widespread racial health equity implications. In subgroup analyses, we found that differences in perceived discrimination during boarding were largely associated with non-Hispanic Black patients. Using the framework of Public Health Critical Race Praxis, we recognize race as a socially and historically derived category that continues to create differential access to resources and power in a society.^11^ We view the differences in race-based experiences as a reflection of the impacts of racism and the historical and ongoing social positioning of non-Hispanic Black individuals in the US.^12^ We also acknowledge that research on racial inequities must move beyond documentation to challenge the mechanisms and power hierarchies that generate them, and we are engaged in additional studies and interventions to address our findings using race-conscious and community-informed frameworks.

Our findings also underscore actionable insights for health care workers to mitigate discrimination during ED boarding. Almost 40% of patients felt inadequately involved in treatment decisions, particularly patients from marginalized groups, who were more likely to report being treated with less courtesy and less respect and as being less intelligent than other patients. More than one-third of all patients felt doctors gave them conflicting information about their care, and more than one-fourth of patients felt that doctors and nurses did not discuss their anxiety or fears about their care. These findings underscore the necessity to examine how racism impacts clinician communication and treatment practices and to develop practices and policies to improve racial health equity in ED boarding.^30^

More than two-thirds of patients were dissatisfied with some element of their care while boarding. Although patient dissatisfaction occurred across all boarding durations, we observed a substantial increase after 24 hours. This agrees with prepandemic evidence that patient experience scores are more favorable among ED sites with shorter boarding times.^10^ Notably, an analysis^10^ using data from 2019 for 78 US EDs found a significant decrease in satisfaction after about 2.5 hours of boarding, far below our cohort’s median boarding time of 15.5 hours. It is unclear whether this difference is due to differences in sample populations, measures of patient satisfaction, or evolving patient expectations of boarding times.

Increasing evidence suggests that racial discrimination and institutionally derived health inequities can be reduced or eliminated. Successful methods include rewriting clinical guidelines with an equity lens,^31^ practitioner activation through timely reminders of institutional racism,^32^ and patient activation to short-circuit physicians’ implicit bias.^33^ Policy interventions at hospital, state, and federal levels are crucial for addressing the structural factors that contribute to discrimination and lower patient satisfaction during ED boarding.

### Limitations

Our study has several limitations. First, it represents the patient experiences at one medical center with its unique demographics, health disparities, and social and historical context. Second, although we met our a priori power calculations, a larger, multicenter cohort could provide more precise estimates and greater power to detect effect modification by race and ethnicity, and separately assess racial and ethnic health inequities for other marginalized groups. Third, our study design was cross-sectional and, thus, cannot determine causality; however, because a trial of boarding time is infeasible, and a cohort study would be prone to recall bias, our approach was determined most appropriate for our study question. Fourth, although we attempted to control for known covariates, we could not control for all relevant factors.

## Conclusions

Our findings suggest that ED boarding longer than 24 hours may increase discrimination and lower patient satisfaction for patients admitted to general medicine services, particularly for patients from marginalized racial and ethnic groups. Priority should be given to interventions at hospital, state, and federal levels to mitigate the national boarding crisis and its potential for disproportionate harm on these patients.

## Notes

Supplement 1. **eAppendix 1.** Discrimination in Medical Settings (DMS) Scale **eAppendix 2.** Adapted Picker Patient Experience (PPE-15) Questionnaire **eTable 1.** Other Self-Reported Races and Ethnicities **eTable 2.** Frequencies and Percentages of Discrimination While Boarding by Race and Ethnicity, by DMS Scale **eTable 3.** Frequencies and Proportions of Dissatisfaction With Care While Boarding by Race and Ethnicity, by Adapted PPE-15 **eTable 4.** Sensitivity Analysis: Associations of Discrimination During ED Boarding (Categorical Race and Ethnicity) **eTable 5.** Sensitivity Analysis: Associations of Dissatisfaction With Care During ED Boarding (Categorical Race and Ethnicity)

Supplement 2.
Data Sharing Statement
