# Supplementary material for: Prolonged Boarding and Racial Discrimination and Dissatisfaction Among Emergency Department Patients
Source: JAMA Netw Open. 2024 Sep 16;7(9):e2433429. doi: 10.1001/jamanetworkopen.2024.33429 (PMC11406394; doi:10.1001/jamanetworkopen.2024.33429)
Supplement: Supplement 1. — eAppendix 1. Discrimination in Medical Settings (DMS) Scale eAppendix 2. Adapted Picker Patient Experience (PPE-15) Questionnaire eTable 1. Other Self-Reported Races and Ethnicities eTable 2. Frequencies and Percentages of Discrimination While Boarding by Race and Ethnicity, by DMS Scale eTable 3. Frequencies and Proportions of Dissatisfaction With Care While Boarding by Race and Ethnicity, by Adapted PPE-15 eTable 4. Sensitivity Analysis: Associations of Discrimination During ED Boarding (Categorical Race and Ethnicity) eTable 5. Sensitivity Analysis: Associations of Dissatisfaction With Care During ED Boarding (Categorical Race and Ethnicity) [file jamanetwopen-e2433429-s001.pdf]

Supplemental Online Content

Olson RM, Fleurant A, Beauparlant S, et al. Prolonged boarding and racial discrimination and dissatisfaction among emergency department patients. *JAMA Netw Open*. 2024;7(9):e2433429. doi:10.1001/jamanetworkopen.2024.33429

eAppendix 1. Discrimination in Medical Settings (DMS) Scale
eAppendix 2. Adapted Picker Patient Experience (PPE-15) Questionnaire
eTable 1. Other Self-Reported Races and Ethnicities
eTable 2. Frequencies and Percentages of Discrimination While Boarding by Race and Ethnicity, by DMS Scale
eTable 3. Frequencies and Proportions of Dissatisfaction With Care While Boarding by Race and Ethnicity, by Adapted PPE-15
eTable 4. Sensitivity Analysis: Associations of Discrimination During ED Boarding (Categorical Race and Ethnicity)
eTable 5. Sensitivity Analysis: Associations of Dissatisfaction With Care During ED Boarding (Categorical Race and Ethnicity)

This supplemental material has been provided by the authors to give readers additional information about their work.

© 2024 Olson RM et al. *JAMA Network Open.*

eAppendix 1. Discrimination in Medical Settings (DMS) Scale

1. How often do you feel you are treated with less courtesy than other people?	Never	Rarely	Sometimes	Most of the time	Always
2. How often do you feel you are treated with less respect than other people?	Never	Rarely	Sometimes	Most of the time	Always
3. How often do you feel you receive poorer service than others?	Never	Rarely	Sometimes	Most of the time	Always
4. How often do you feel you doctor or nurse acts as if he or she thinks you are not smart?	Never	Rarely	Sometimes	Most of the time	Always
5. How often do you feel your doctor or nurse acts as if he or she is afraid of you?	Never	Rarely	Sometimes	Most of the time	Always
6. How often do you feel your doctor or nurse acts as if he or she is better than you?	Never	Rarely	Sometimes	Most of the time	Always
7. How often do you feel you feel like a doctor or nurse is not listening to what you were saying?	Never	Rarely	Sometimes	Most of the time	Always

© 2024 Olson RM et al. *JAMA Network Open.*

eAppendix 2. Adapted Picker Patient Experience (PPE-15) Questionnaire

Please reflect on your experience while boarding in the Emergency Room during this current hospitalization.

1. When you had important questions to ask a doctor, did you get answers that you could understand?

Yes, always/Yes, sometimes/No/I had no need to ask

2. When you had important questions to ask a nurse, did you get answers that you could understand?

Yes, always/Yes, sometimes/No/I had no need to ask

3. Sometimes in a hospital, one doctor or nurse will say one thing and another will say something quite different. Did this happen to you?

Yes, often/Yes, sometimes/No

4. If you had any anxieties or fears about your condition or treatment, did a doctor discuss them with you?

Yes, completely/Yes, to some extent/No/I didn't have any anxieties or fears

5. Did doctors talk in front of you as if you weren't there?

Yes, often/Yes sometimes/No

6. Did you want to be more involved in decisions made about your care and treatment?

Yes, definitely/Yes, to some extent/No

7. Overall, did you feel you were treated with respect and dignity while you were boarding in the Emergency Room?

Yes, always/Yes, sometimes/No

8. If you had any anxieties or fears about your condition or treatment, did a nurse discuss them with you?

Yes, completely/Yes, to some extent/No/I didn't have any anxieties or fears

9. Did you find someone on the hospital staff to talk to about your concerns?

Yes, definitely/Yes, to some extent/No/I had no concerns

10. Were you ever in pain?

Yes/No

If yes…

Do you think the hospital staff did everything they could to help control your pain?

Yes, definitely/Yes, to some extent/No

13. Did a member of staff explain the purpose of the medicines you were to take in a way you could understand?

© 2024 Olson RM et al. *JAMA Network Open.*

Yes, completely/Yes, to some extent/No/I didn't need an explanation/I had no medicines

14. Did a member of staff tell you about medication side effects to watch for?

Yes, completely/Yes, to some extent/No/I didn't need an explanation

© 2024 Olson RM et al. *JAMA Network Open.*

eTable 1. Other Self-Reported Races and Ethnicities

	n
African	3
"All of the races"	1
American	1
Armenian	1
Asian	11
Brazilian	1
Cape Verdean	1
Cuban	1
Ethiopian	1
Haitian	1
Irish	1
Jewish	1
Mediterranean	1
Middle Eastern	3
Multiracial	3
Portuguese	1
Puerto Rican	1
Native American	3
Native West Indian	1
Total	37

© 2024 Olson RM et al. *JAMA Network Open.*

eTable 2. Frequencies and Percentages of Discrimination While Boarding by Race and Ethnicity, by DMS Scale

	**Total, N**	**Non-Hispanic White**	**Marginalized race and ethnicity**	NH Black	Hispanic	Other	P value[1]
**Reason for experience of discrimination**							
Treated with less courtesy, No. (%)	94 (17.9)	31 (12.6)	62 (22.6)	39 (26.2)	13 (14.8)	10 (27.0)	**.003**
Treated with less respect, No. (%)	82 (15.7)	26 (10.6)	54 (19.7)	29 (19.5)	14 (15.9)	11 (29.7)	**.004**
Received poorer service, No. (%)	74 (14.1)	28 (11.4)	44 (16.1)	27 (18.1)	10 (11.4)	7 (18.9)	.12
Medical staff treated me as less smart, No. (%)	82 (15.7)	28 (11.4)	53 (19.4)	33 (22.3)	10 (11.4)	10 (27.0)	**.01**
Medical staff acted afraid of me, No. (%)	21 (4.0)	8 (3.3)	13 (4.7)	10 (6.7)	0 (0)	3 (8.11)	.39
Medical staff acted better than me	62 (11.8)	29 (11.8)	33 (12.0)	21 (14.1)	5 (5.7)	7 (18.9)	.93
Medical staff didn't listen to me, No. (%)	112 (21.3)	54 (22.0)	56 (20.4)	32 (21.5)	13 (14.8)	11 (29.7)	.67

1. Comparing NH White vs. marginalized race and ethnicity

© 2024 Olson RM et al. *JAMA Network Open.*

eTable 3: Frequencies and Proportions of Dissatisfaction With Care While Boarding by Race and Ethnicity, by Adapted PPE-15

	Total	NH White	Marginalized race and ethnicity	NH Black	Hispanic	Other	P value
**Information and education**							
Doctors' answers to questions not clear	91 (18.1)	42 (17.1)	49 (17.9)	28 (18.8)	12 (13.6)	9 (24.3)	0.81
Nurses' answers to questions not clear	78 (14.9)	33 (13.4)	42 (15.3)	25 (16.8)	9 (10.2)	8 (21.6)	0.54
**Coordination of care**							
Doctors gave conflicting information	189 (36.0)	81 (32.9)	107 (39.1)	65 (43.6)	30 (34.1)	12 (32.4)	0.15
Nurse gave conflicting information	98 (18.7)	41 (16.7)	55 (20.1)	31 (20.8)	14 (15.9)	10 (27.0)	0.32
**Physical comfort**							
Staff didn't do enough to control pain	101 (19.2)	50 (20.3)	48 (17.5)	31 (20.8)	6 (6.8)	11 (29.3)	0.41
**Emotional support**							
Doctor didn't discuss my anxieties/fears	136 (25.9)	67 (27.2)	66 (24.1)	41 (27.5)	17 (19.3)	8 (21.6)	0.41
Nurse didn't discuss my anxieties/fears	136 (25.9)	65 (26.4)	68 (24.8)	40 (26.9)	20 (22.7)	8 (21.6)	0.68
Not easy to find someone to discuss concerns	121 (23.1)	57 (23.2)	62 (22.6)	33 (22.2)	20 (22.7)	9 (24.3)	0.88
**Respect for patient preferences**							
Doctors sometimes talked as if I wasn't there	59 (11.2)	25 (10.2)	34 (12.4)	20 (13.4)	9 (10.2)	5 (13.5)	0.42

© 2024 Olson RM et al. *JAMA Network Open.*

Not always treated with respect or dignity	72 (13.7)	28 (11.4)	42 (15.3)	25 (16.8)	9 (10.2)	8 (21.6)	0.19
Not sufficiently involved in decisions about treatment and care	202 (38.5)	72 (29.3)	128 (46.7)	62 (41.6)	45 (51.1)	21 (56.8)	**<.001**
**Continuity and transition**							
Purpose of medicines not explained	52 (9.9)	22 (8.9)	29 (10.6)	18 (12.1)	4 (4.6)	7 (18.9)	0.53
Side effects of medicines not explained	119 (24.1)	62 (27.7)	55 (20.6)	32 (21.6)	12 (14.5)	10 (30.6)	0.07

© 2024 Olson RM et al. *JAMA Network Open.*

eTable 4: Sensitivity Analysis: Associations of Discrimination During ED Boarding (Categorical Race and Ethnicity)

	White		Black		Hispanic	
**Boarding Time (hrs)**	aOR (95%)	P value	aOR (95%)	P value	aOR (95%)	P value
<4	1 [Reference]		1 [Reference]		1 [Reference]	
4-24	.95 (0.45-2.00)	0.89	1.08 (.43-2.71)	0.86	2.91 (.62-13.54)	0.17
>24	1.65 (0.79- 3.42)	0.18	3.00 (1.18-7.59)	0.02	3.12 (0.66-14.67)	0.15

eTable 5: Sensitivity Analysis: Associations of Dissatisfaction With Care During ED Boarding (Categorical Race and Ethnicity)

	White		Black		Hispanic	
**Boarding Time (hrs)**	aOR (95%)	P value	aOR (95%)	P value	aOR (95%)	P value
<4	1 [Reference]		1 [Reference]		1 [Reference]	
4-24	1.13 (0.55-2.32)	0.75	1.51 (0.60-3.81)	0.38	0.30 (0.05-1.72)	0.18
>24	1.68 (0.79-3.60)	0.18	5.11 (1.59-16.4)	0.006	0.33 (0.06-1.9)	0.22

© 2024 Olson RM et al. *JAMA Network Open.*
