# Supplementary material for: Prolonged Boarding and Racial Discrimination and Dissatisfaction Among Emergency Department Patients
Source: JAMA Netw Open. 2024 Sep 16;7(9):e2433429. doi: 10.1001/jamanetworkopen.2024.33429 (PMC11406394; doi:10.1001/jamanetworkopen.2024.33429)
Supplement: Supplement 2. — Data Sharing Statement [file jamanetwopen-e2433429-s002.pdf]

Data Sharing Statement

Olson. Prolonged Boarding and Racial Discrimination and Dissatisfaction Among Emergency Department Patients. *JAMA Netw Open*. Published September 16, 2024. doi:10.1001/jamanetworkopen.2024.33429

Data
Data available: No
